# Hippocampal transcriptome-wide association study and pathway analysis of mitochondrial solute carriers in Alzheimer’s disease

**DOI:** 10.1038/s41398-024-02958-0

**Published:** 2024-06-10

**Authors:** Jing Tian, Kun Jia, Tienju Wang, Lan Guo, Zhenyu Xuan, Elias K. Michaelis, Russell H. Swerdlow, Alzheimer’s Disease Neuroimaging Initiative, Heng Du

**Affiliations:** 1 Department of Pharmacology and Toxicology, University of Kansas, Lawrence, KS USA; 2 Department of Biological Sciences, The University of Texas at Dallas, Richardson, TX USA; 3 Department of Pharmaceutical Chemistry, University of Kansas, Lawrence, KS USA; 4 Alzheimer’s Disease Research Center, University of Kansas Medical Center, Kansas City, KS USA

**Keywords:** Clinical genetics, Clinical genetics

## Abstract

The etiopathogenesis of late-onset Alzheimer’s disease (AD) is increasingly recognized as the result of the combination of the aging process, toxic proteins, brain dysmetabolism, and genetic risks. Although the role of mitochondrial dysfunction in the pathogenesis of AD has been well-appreciated, the interaction between mitochondrial function and genetic variability in promoting dementia is still poorly understood. In this study, by tissue-specific transcriptome-wide association study (TWAS) and further meta-analysis, we examined the genetic association between mitochondrial solute carrier family (*SLC25*) genes and AD in three independent cohorts and identified three AD-susceptibility genes, including *SLC25A10*, *SLC25A17*, and *SLC25A22*. Integrative analysis using neuroimaging data and hippocampal TWAS-predicted gene expression of the three susceptibility genes showed an inverse correlation of *SLC25A22* with hippocampal atrophy rate in AD patients, which outweighed the impacts of sex, age, and apolipoprotein E4 (*ApoE4*). Furthermore, *SLC25A22* downregulation demonstrated an association with AD onset, as compared with the other two transcriptome-wide significant genes. Pathway and network analysis related hippocampal *SLC25A22* downregulation to defects in neuronal function and development, echoing the enrichment of *SLC25A22* expression in human glutamatergic neurons. The most parsimonious interpretation of the results is that we have identified AD-susceptibility genes in the *SLC25* family through the prediction of hippocampal gene expression. Moreover, our findings mechanistically yield insight into the mitochondrial cascade hypothesis of AD and pave the way for the future development of diagnostic tools for the early prevention of AD from a perspective of precision medicine by targeting the mitochondria-related genes.

## Introduction

Late-onset Alzheimer’s disease (AD) is aheterogeneous neurodegenerative disorder symptomatically defined by gradual cognitive decline [1, 2]. Despite a consensus that hippocampal dysfunction constitutes a pivotal biological basis for AD-related cognitive deficits as well as being a critical predictor of AD risk [2–6], the detailed mechanisms of hippocampal vulnerability in this neurodegenerative disorder thus far remain elusive. The etiopathogenesis of AD is proposed to be a perplexing combination of aging factors, protein aggregates, brain dysmetabolism, and genetic risks [7–9]. In addition to the deleterious influence of AD-associated toxic molecules, including amyloid beta (Aβ) and pathological tau, basic and clinical studies have accentuated a mitochondrial pathway of hippocampal pathology and thus the resulting cognitive deficits in AD [9–13]. Although the polygenic nature of AD pathogenesis has been increasingly recognized [14–17], the interaction between mitochondrial function and genetic variability in the development of AD remains understudied.

Mitochondrial solute carriers, also known as solute carrier family 25 (SLC25) spanning from SLC25A1 to SLC25A53, constitute a mitochondria-specific sub-family of solute carriers (SLCs) that transports a plethora of substrates across the mitochondrial membrane [18, 19]. The substrate repertoire of SLC25 includes a collection of molecules encompassing inorganic ions, nucleotides, amino acids, and enzyme cofactors, as well as Krebs cycle metabolites [19–22]. The SLC25 family composed of a collection of members typically feature three homologous repeats and structure into three distinct regions: the cytoplasmic gate, the substrate binding site, and the matrix gate [23]. Depending on the concentrations of transported substrates, counter-substrates, competing substrates, or interactions with allosteric inhibitors or activators, the substrate binding site of SLC25 family members opens to the intermembrane space in the cytoplasmic state (c-state) and rotates to the matrix side in the matrix state (m-state) to regulate substrate transport direction [24, 25]. Most of the SLC25 family isoforms exhibit widespread expression across various tissues, whereas some isoforms demonstrate tissue-specific expression patterns to accommodate cell- and tissue-specific functions such as the abundance of UCP1 carrier SLC25A7 in the brown adipose tissue, and UCP4 carrier SLC25A27 in the brain [18]. The importance of these SLC25 substrates to mitochondrial biology and the abundance of a full spectrum of SLC25 family members in the brain, including the hippocampus, together establish a pivotal role of SLC25 in brain physiology and further underscore a causal relationship between SLC25 mutations and multiple neurological disorders such as thiamine metabolism dysfunction syndrome 4, combined D-2- and L-2-hydroxyglutaric aciduria, mitochondrial phosphate carrier deficiency, early infantile epileptic encephalopathy, and many others [26–31]. The growing supportive evidence of hippocampal mitochondrial dysfunction in AD has thus raised a critical and yet-understudied scientific question of whether the gene traits of mitochondrial SLCs may predispose hippocampal lesions, promoting the development of this neurodegenerative disorder.

In recent decades, genetic variants have been associated with AD by genome-wide association studies (GWAS) [14–17], shedding light on the diagnosis, prevention, and management of AD from a perspective of precision medicine. Transforming this idea into reality, however, is stalled by obstacles in interpreting the influence(s) of GWAS-identified single nucleotide polymorphisms (SNPs) on transcriptomics and subsequently, proteomic landscapes. Owing to the recent progress in deep- and machine-learning gene expression imputation methods, tissue-specific transcriptome-wide association study (TWAS) is emerging as a critical supplement to GWAS in bridging the disconnect between gene expression and disease traits. By using the reference panels for gene expression in different tissues, TWAS incorporates regulatory weights of genetic variants to impute gene expression in specific tissues for the prediction of gene-trait association [32–34]. This method offers opportunities to gain insights into the biological consequences of gene polymorphisms and enables our examination of *SLC25*-associated traits in AD.

In the current study, we aimed to examine the association between regulatory variants of *SLC25* family genes with AD risk and hippocampal pathology. We adopted whole-genome sequencing (WGS) GWAS summary statistics from two large-scale discovery cohorts and individual WGS data from the validation cohort, the Alzheimer’s Disease Neuroimaging Initiative (ADNI) cohort. In reference to the Genotype-Tissue Expression (GTEx) Consortium atlas of genetic regulatory effects [35], we performed hippocampal TWAS [36] for AD-associated genes in the *SLC25* family and identified three transcriptome-wide significant genes including *SLC25A10*, *SLC25A17*, and *SLC25A22* among the *SLC25* family. In further examination of the gene-trait relationship among the three studied genes, downregulation of *SLC25A22* was associated with accelerated hippocampal atrophy in AD patients and increased hazard of dementia. Lastly, functional annotation mounted the effects of *SLC25A22* regulatory variants to multiple neuronal development- and function-related pathways. Collectively, in this study, we have identified AD-susceptibility genes in the *SLC25* family by TWAS-predicted hippocampal gene expression and further gene-trait association analysis. The results add supportive evidence to the mitochondrial pathway of AD and have positive impacts on future precision medicine targeting mitochondria-related *SLC25* genes, especially *SLC25A22*, for the diagnosis and early prevention of AD in a subset of patients.

## Materials and Methods

### Participants and genetic data sources

Data from the participants were obtained from multiple sources, including two discovery cohorts: cohort 1 GWAS summary statistics downloaded from the Alzheimer disease (AD) GWAS Catalog on 11/27/2023 for study “GCST013197” with 90,338 AD cases and 1,036,225 nonAD controls [14], as well as cohort 2 freely-accessible GWAS summary data for Alzheimer’s disease cohorts with 71,880 AD cases and 383,378 nonAD controls [37]. Original data of the validation cohort, including WGS data, neuroimaging data, and patient information used in the preparation of this article, were obtained from the Alzheimer’s Disease Neuroimaging Initiative (ADNI) database (adni.loni.usc.edu). ADNI was launched in 2003 as a public-private partnership, led by Principal Investigator Michael W. Weiner, MD. The primary goal of ADNI has been to test whether serial magnetic resonance imaging (MRI), positron emission tomography (PET), other biological markers, and clinical and neuropsychological assessments can be combined to measure the progression of mild cognitive impairment (MCI) and early Alzheimer’s disease (AD). For up-to-date information, see www.adni-info.org.

### TWAS analysis using GWAS summary statistics

GWAS summary statistics of the two discovery cohorts were analyzed by transcriptome-wide association study (TWAS) to establish the connection between phenotypic associations of SNPs and gene expression levels. In this study, we employed the TIGAR-V2 tool for conducting TWAS analysis [36]. Bayesian DPR cis-eQTL weights for the hippocampus and reference LD covariance for chromosomes 1-22 based on GTEx V8 data were obtained from SYNAPSE (project SynID: syn16804286). TWAS analysis of summary statistics for cohort 1 directly employed the z score, while for cohort 2, SNP z scores were converted from odds ratio (OR), the lower bound of the 95% confidence interval (CI), and standard error (SE) using the following equation:

$$Z\,{score}=\frac{\log \left({OR}\right)-\log \left({CI}\right)}{1.96* {SE}}$$

We conducted TWAS summary analysis on a genomic region spanning 1 million base pairs around the gene (± 1 million base pairs flanking the gene region) and set the weight threshold at 0. For dichotomous phenotypes, logistic regression was selected. We chose the statistical method employed by S-PrediXcan for the burden Z test to complement the TWAS individual analysis method.

### TWAS analysis for individual genotyping data

The original WGS data from the ADNI cohort underwent an initial liftover process, transitioning from human genome version 19 (hg19) to Genome Reference Consortium Human Build 38 (GRCh38). We subsequently conducted TWAS analysis utilizing the PrediXcan software [38]. Consistent with the TWAS summary statistics analysis, we here used hippocampal Bayesian DPR cis-eQTL weights from chromosomes 1-22 as a reference database to calculate the effect size of genes. In alignment with the analysis of TWAS summary statistics, Bayesian DPR eQTL weights were employed as the reference database for gene expression predictions. Disease association analysis was executed on a cohort comprised of 229 individuals with AD and 246 nonAD healthy controls. Logistic regression was selected for dichotomous univariate phenotypes.

### Neuroimaging association analysis

Individuals from 65 to 95 years of age, who had multiple magnetic resonance imaging (MRI)-based hippocampal volumetric measurements within a 2-year period of the last visit, with an interval between two MRI scans of at least 9 months in the ADNI cohort were included in calculations of the relationship between genetic regulation of susceptibility genes and the atrophy rate of the left, right, and total hippocampus (in percentage). Individuals with hippocampal MRI scans that failed to pass the quality check on their last visit were removed from the study. Hippocampal measurement data from the University of California, San Francisco (UCSF)-cross-sectional FreeSurfer 5.1 was used for the analysis [39–45]. The annualized hippocampal atrophy rate was calculated using the following equation:

$${Hippocampal\; atrophy\; rate}=\frac{(-\Delta {hippocampal\; volume})* 12* 100}{{\rm{Baseline\; hippocampal\; volume}}* \Delta {months}}$$

Weighted least square regression (WLSR) analysis was used to investigate the correlation between hippocampal atrophy rate and the effect sizes of the regulatory variants of the susceptibility genes. Graphs were generated using R studio.

### Functional annotation of the identified genes

QIAGEN Ingenuity Pathway Analysis (QIAGEN IPA) web service was used to examine the canonical pathways and core analysis of variants (loss and gain) related to the identified genes. Comparison analysis of genes with opposite regulation (z score) between subjects with *SLC25A22* up- and down-regulation was performed to identify the differential pathways related to disease and functions also using Qiagen IPA. Hippocampal gene network analysis to examine the involvement of the identified genes with opposite regulation (z score) between subjects with *SLC25A22* up- and downregulation in cohesive gene clusters was conducted by using the HumanBase (https://hb.flatironinstitute.org) [46].

### Human single-cell RNA sequencing data

To investigate the expression patterns of *SLC25A18* and *SLC25A22*, we leveraged open-access human RNA-Seq data available through the Cell Types Database on ALLEN BRAIN MAP. Datasets, including M1 10X genomics, Multiple Cortical Areas-smart-seq, and MTG 10X Seattle Alzheimer’s Disease Brain Cell Atlas (SEA-AD) studies from the “Human Multiple Cortical Areas” protocol, were obtained from the specified website (https://portal.brain-map.org/atlases-and-data/rnaseq). Data generation was supported by multiple awards, including the Brain Initiative Cell Census Network (BICCN) award U01MH114812 from the National Institute of Mental Health and the National Institute of Neurological Disorders and Stroke, and by the Allen Institute for Brain Science [47, 48]. The expression profiles of *SLC25A18* and *SLC25A22*, represented by trimmed mean values, were retrieved from the three mentioned databases and subsequently visualized using R Studio.

### Statistical analysis and meta-analysis

All data were analyzed using SPSS statistical software (version 29.0.0.0; IBM, Armonk, NY) unless otherwise indicated. The two-tailed Student’s t-test was employed to compare means between the two groups. Samples were normal distribution and of similar variance between the groups that are being statistically compared. Chi-squared and Fisher’s exact probability tests were used for the analysis of qualitative data differences. The meta-analysis of TWAS statistics from the two discovery cohorts and the ADNI cohort was performed with the use of the weighted Fisher’s method (wFisher) in metapro R package [49]. The *p* values from the TWAS analysis, corresponding sample sizes, and effect direction were input for this meta-analytical approach for the calculation of combined *p* values. Genes with a combined *p* value of less than 0.05 in the meta-analysis in the studied three cohorts were considered transcriptome-wide significant genes. The transcriptome-wide significant genes with a combined *p* value of less than 0.01 were considered as top candidate genes for further gene-trait analysis. Weighted least squares regression (WLSR) was used to examine the correlation between hippocampal atrophy rate and magnitude of genetic regulation. Partial least squares regression (PLSR) analysis was used to predict the influence of critical co-variables, including age (younger than 80 vs older than 80), sex (female vs male), and *ApoE4* status (carrier vs non-carrier) as well as genetic regulation on the development of hippocampal lesions in small-scale cohort (*n* < 200) [50, 51]. Multivariable Cox proportional hazards models were used to examine the time-event relationship between the time point of event and covariates, including genetic regulation of susceptibility genes (downregulated vs. upregulated), age (age at the initial visit), sex (female vs. male), and *ApoE4* status (carrier vs. noncarrier). In this time-event analysis, an event was defined as a global clinical dementia rating (CDR) score of 1 or higher within the follow-up period of up to ten years. The time point (year) of the CDR score of 1 was noted as “time of event”. Subjects with a CDR score of 0 or 0.5 by the end of the ten-year observation period were noted as “no event”. Statistical significance was set at *p* < 0.05 (two-sided).

## Results

### Identification of AD-associated SLC25 family genes with hippocampal TWAS analysis

**Fig. 1:**
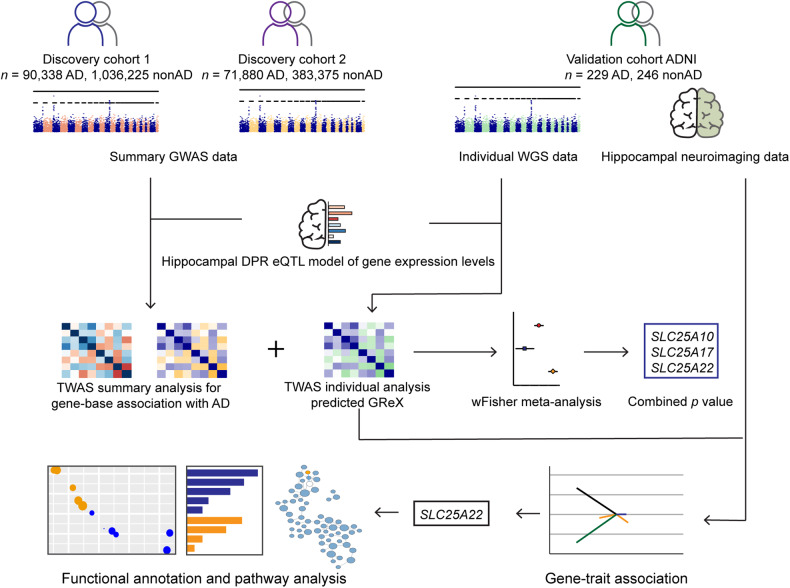
Schematic graph of the study design. GWAS: genome-wide association studies, WGS whole genome sequencing, ADNI the Alzheimer’s Disease Neuroimaging Initiative, eQTL expression quantitative trait loci, DPR Dirichlet process regression. GReX genetically regulated gene expression. wFisher weight Fisher method.

**Fig. 2:**
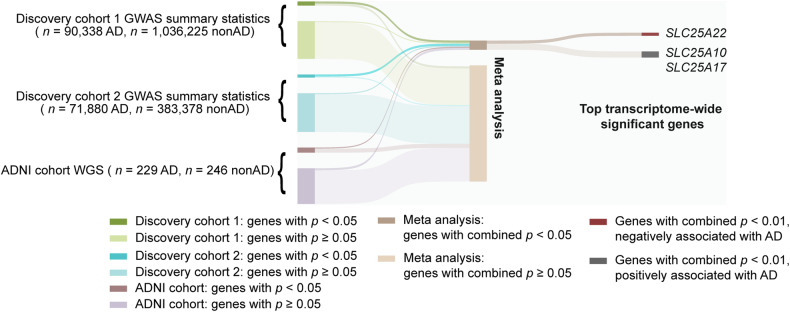
Flowchart of the meta-analysis. Each cohort is grouped based on the gene *p* value at a threshold of 0.05. Discovery cohort 1, *n* = 90,338 AD cases, *n* = 1,036,225 nonAD controls; discovery cohort 2, *n* = 71,880 AD cases, *n* = 383,378 nonAD controls; ADNI cohort, *n* = 229 AD cases, *n* = 246 nonAD controls. Genes including *SLC25A10*, *SLC25A17*, and *SLC25A22* with a combined *p* value less than 0.01 via meta-analysis were selected for phenotype association study.

To integrate the WGS data with tissue-specific reference transcriptomic data for gene-based association studies, we performed hippocampal TWAS using Bayesian Dirichlet process regression (DPR) expression quantitative trait loci (eQTL) weights from the GTEx V8 reference [36]. A schematic workflow description of the TWAS analysis and further phenotype association is shown in Fig. 1. Our discovery cohorts consisted of two extensive datasets: the “GCST013197” study from the GWAS Catalog, encompassing 90,338 AD cases and 1,036,225 nonAD controls, and a freely-accessible Alzheimer’s disease cohort with 71,880 AD cases and 383,378 nonAD controls [14, 37]. GWAS summary statistics of the two discovery cohorts were subjected to hippocampal TWAS using the TIGAR-V2 tool to predict the effects of regulatory variants on gene expression in the hippocampus in reference to hippocampal SNP-expression associations. The TWAS summary-burden Z test results, quantified with the S-Predixcan calculation method, indicated that among the genes with predicted values, forty-one genes out of the fifty-three *SLC25* family members were identified in the two discovery cohorts. Given the availability of neuroimaging data for further gene-trait analysis, we chose the Alzheimer’s Disease Neuroimaging Initiative (ADNI) cohort for validation. The individual WGS data from the ADNI cohort (Table 1) underwent TWAS individual analysis using Predixcan software, employing the same reference as the mentioned TWAS summary analysis, to predict the regulation of genes, including the *SLC25* family genes. We next utilized the weighted Fisher’s method (wFisher) to perform meta-analysis based on the TWAS statistics from two discovery cohorts and the ADNI cohort as validation to enhance statistical power for the combined *p* values [49]. At the nominal threshold of *p* value at 0.01, three AD-associated genes, *SLC25A10*, *SLC25A17*, and *SLC25A22* were determined as candidate genes for further gene-to-disease trait association analysis (Fig. 2 and Table 2). The results showed that the upregulation of *SLC25A10*, which encodes the mitochondrial dicarboxylate carrier [52], and *SLC25A17*, which encodes the peroxisomal transporter for multiple nucleotides [53], are positively associated with AD (Fig. 2 and Table 2). In contrast, the genetic regulation of *SLC25A22*, which encodes the mitochondrial glutamate carrier [54], is in a negative relationship with AD (Fig. 2 and Table 2). In contrast, no disease association between genetic regulation of *SLC25A10, SLC25A17*, or *SLC25A22* was determined in the cerebellum by TWAS statistics and meta-analysis at the nominal threshold of *p* value at 0.01 (Supplementary Table 1). These findings corroborate previous observations of brain region-related transcriptomic difference in humans, including brain aging and AD subjects [55–57].

**Table 1:** Demographics of the ADNI cohort.

		nonAD (*n* = 246)	AD (*n* = 229)	*p* value
	Age^a^	78.3 ± 0.92	79.11 ± 0.98	0.2326
	Education years^a^	16.6 ± 0.33	15.98 ± 0.37	0.0134
	Sex % female	54.48%	39.74%	0.0013
	*ApoE4*% carrier	26.42%	65.50%	<0.0001
Race	American Indian/Alaska Native	0.41%	0%	0.0568
	Asian	0.81%	1.75%	
	Black or African American	5.28%	1.31%	
	Other	1.63%	0.44%	
	White	91.87%	96.50%	
AD symptom control medication	None	96.34%	24.02%	<0.0001
	ACHEI	3.25%	29.26%	
	Memantine	0.41%	8.29%	
	ACHEI + memantine	0%	38.43%	
Lifestyle	Alcohol use	3.25%	0.87%	0.0712
	Substance use	0.81%	0%	0.1715
	Smoking	35.37%	37.55%	0.6204

^a^Data represented by mean ± 95% CI or percentage.

Two-tailed Student’s t-test were used to compare the difference for quantitative variables, Chi-squared and Fisher’s exact probability tests for qualitative variables.

**Table 2:** Association of *SLC25* genes with AD.

Gene Symbol	Discovery cohort 1		Discovery cohort 2		ADNI		Meta analysis	
	z score	*p* value	z score	*p* value	z score	*p* value	Combined effect direction	Combined *p* value
*SLC25A1*	0.5077	0.6117	0.1698	0.8652	1.4016	0.1610	+	0.7450
*SLC25A2*	-0.7040	0.4815	0.0427	0.9659	-2.0340	0.0419	-	0.6916
*SLC25A3*	1.6519	0.0985	-0.2983	0.7654	-0.4681	0.6397	+	0.2242
*SLC25A4*	-0.2321	0.8165	-1.9559	0.0505	-1.2302	0.2186	+	0.1460
***SLC25A10***	2.1248	0.0336	1.8296	0.0673	-0.1660	0.8681	+	0.0087**
*SLC25A11*	0.0586	0.9533	0.9431	0.3457	1.5647	0.1177	+	0.6195
*SLC25A12*	0.7477	0.4547	-0.0421	0.9664	-1.2909	0.1967	-	0.6621
*SLC25A13*	-0.7608	0.4468	0.0334	0.9734	0.4254	0.6706	-	0.6507
*SLC25A15*	-0.6999	0.4840	0.9119	0.3618	-0.9747	0.3297	+	0.9136
*SLC25A16*	0.9116	0.3620	0.3355	0.7372	0.5582	0.5767	+	0.4464
***SLC25A17***	1.7265	0.0843	2.4296	0.0151	1.0219	0.3068	+	0.0060**
*SLC25A18*	-0.0112	0.9911	0.8669	0.3860	1.1946	0.2322	-	0.6955
*SLC25A19*	-0.2900	0.7718	-1.1711	0.2416	-2.1486	0.0317	+	0.4089
*SLC25A20*	0.2085	0.8349	0.4456	0.6559	0.8888	0.3741	+	0.8074
*SLC25A21*	0.5333	0.5938	1.6276	0.1036	0.9696	0.3322	+	0.1787
***SLC25A22***	-2.5862	0.0097	-2.0767	0.0378	-2.2285	0.0258	-	0.0017**
*SLC25A23*	-0.0521	0.9584	-0.2862	0.7747	-2.1386	0.0324	-	0.9844
*SLC25A24*	-0.1245	0.9009	-0.1503	0.8805	1.0348	0.3008	+	1.0000
*SLC25A25*	0.0952	0.9242	0.6740	0.5003	-0.7369	0.4612	-	0.7508
*SLC25A26*	0.5809	0.5613	-0.1516	0.8795	-0.9703	0.3319	+	0.8130
*SLC25A27*	1.3758	0.1689	0.6076	0.5435	0.7612	0.4465	-	0.1920
*SLC25A28*	-1.3971	0.1624	-2.3004	0.0214	1.1392	0.2546	+	0.0151*
*SLC25A29*	1.9130	0.0557	0.2128	0.8315	-0.1532	0.8783	-	0.1031
*SLC25A30*	0.0997	0.9206	-0.7293	0.4658	-0.3294	0.7419	+	0.8212
*SLC25A31*	1.0824	0.2791	0.6781	0.4977	-0.9561	0.3390	-	0.2757
*SLC25A32*	-0.9635	0.3353	-0.2995	0.7646	-1.1614	0.2455	+	0.4300
*SLC25A33*	0.0847	0.9325	0.9105	0.3625	0.5456	0.5853	+	0.6254
*SLC25A34*	1.5952	0.1107	1.1030	0.2700	-0.0594	0.9526	-	0.0787
*SLC25A35*	-2.1594	0.0308	-0.5169	0.6052	-1.8372	0.0662	+	0.0481*
*SLC25A36*	0.6008	0.5480	0.3419	0.7324	2.6194	0.0088	+	0.6138
*SLC25A37*	-0.0633	0.9495	-1.7262	0.0843	0.6879	0.4915	-	0.2457
*SLC25A38*	-1.4214	0.1552	-1.7153	0.0863	-0.5464	0.5848	-	0.0441*
*SLC25A39*	-0.6308	0.5282	-0.4812	0.6304	-1.5664	0.1173	-	0.5470
*SLC25A40*	0.2196	0.8262	0.2281	0.8196	0.8200	0.4122	+	0.9071
*SLC25A41*	-2.0160	0.0438*	-1.5594	0.1189	-0.7124	0.4762	-	0.0177*
*SLC25A42*	0.5720	0.5674	-0.0206	0.9836	-0.3883	0.6978	+	0.7740
*SLC25A44*	0.1422	0.8869	1.2463	0.2127	0.3264	0.7441	+	0.4278
*SLC25A45*	-1.0989	0.2718	-0.6714	0.5019	1.5998	0.1096	-	0.2713
*SLC25A46*	0.0536	0.9573	0.4119	0.6804	-0.3326	0.7394	+	0.9175
*SLC25A48*	-1.1837	0.2366	-0.6966	0.4860	-0.1246	0.9009	-	0.2358

The bold values are genes with combined *p* value lower than 0.01.

* combined *P* < 0.05, ** combined *P* < 0.01.

### Association of susceptibility SLC25 family genes with hippocampal atrophy in AD

**Fig. 3:**
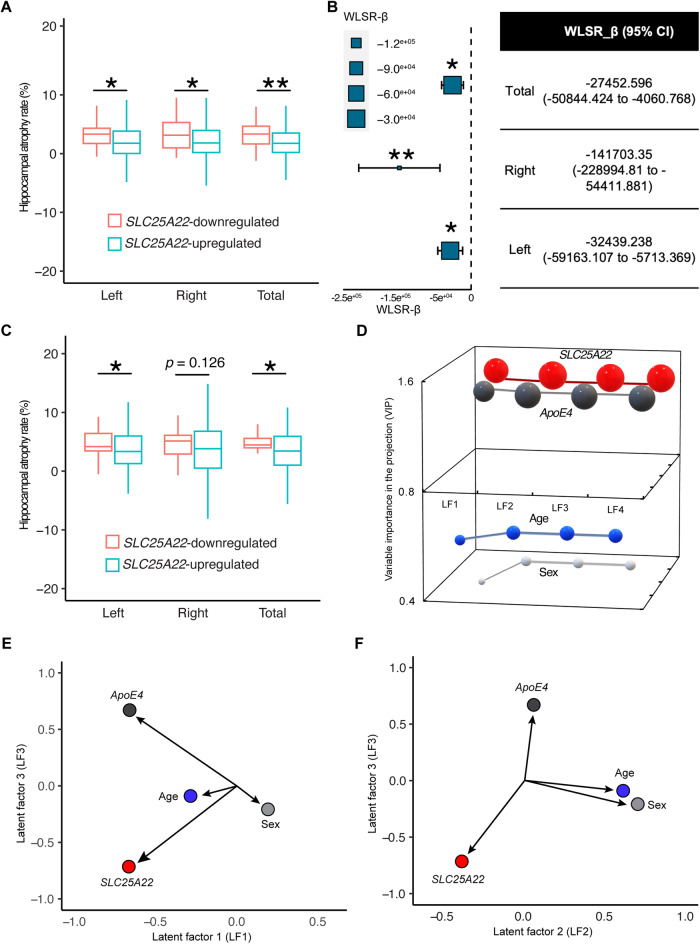
Analysis of the relationship between hippocampal *SLC25A22* genetic regulation and hippocampal atrophy rate. **A** Comparison of hippocampal atrophy rate between *SLC25A22* down- and up-regulated subjects in the AD and nonAD combined cohort. Two-tail student t-test. *SLC25A22* downregulated *n* = 38, *SLC25A22* upregulated *n* = 222. * *p* < 0.05, ** *p* < 0.01. Data is represented as mean ± 95% CI. **B** Weighted least square regression (WLSR) analysis for the correlation between hippocampal atrophy rate and the effect size of *SLC25A2*2 genetic regulation. *SLC25A22* downregulated *n* = 38, *SLC25A22* upregulated *n* = 222. * *p* < 0.05, ** *p* < 0.01. **C** Comparison of hippocampal atrophy rate between *SLC25A22* down-and up-regulated subjects in AD patients. Two-tail student t-test. *SLC25A22* downregulated *n* = 17, *SLC25A22* upregulated *n* = 79. * *p* < 0.05. Data is represented as mean ± 95% CI. **D**–**F** Partial least square regression (PLSR) analysis in the AD cohort. The annualized hippocampal atrophy rate was set as the dependent variable. *SLC25A22*, age, sex, and *ApoE4* status were input as covariables in the analysis. **D** Variable importance in the projection (VIP) of *SLC25A22*, age, sex, and *ApoE4*. **E**, **F** GGraphics of latent factors 1 and 3 (**E**) as well as 2 and 3 (**F**).

Hippocampal atrophy is a characteristic pathology of AD and accelerated volumetric loss of the hippocampus correlates with cognitive deficits associated with disease progression [58, 59]. To this end, it would be of great interest to examine the relationship between transcriptome-wide significant genes—including *SLC25A10*, *SLC25A17*, and *SLC25A22*—and hippocampal atrophy to establish the gene-trait association. The annualized rate of hippocampal atrophy was calculated in subjects with multiple MRI scans and MRI-based hippocampal volumetric measurements within 2 years of the last visit at an interval of at least 9 months between two MRI scans in the ADNI cohort (Table 3). In contrast to the lack of association between genetic regulation of *SLC25A10* (Supplementary Fig. 1A & B) or *SLC25A17* (Supplementary Fig. 2A & B) and annualized hippocampal loss, subjects with downregulation of hippocampal *SLC25A22* demonstrated increased annualized loss of the left, right, and total (Fig. 3A) hippocampal volume as compared to those with *SLC25A22* upregulation. Further weighted least square regression (WLSR) analysis showed a negative correlation between hippocampal atrophy rate and the magnitude of *SLC25A2*2 genetic regulation (Fig. 3B). In the cohort of AD patients only, accelerated hippocampal atrophy was also associated with hippocampal *SLC25A22* downregulation (Fig. 3C). Such an inverse relationship between hippocampal gene expression and hippocampal atrophy rate was not detected with *SLC25A10* (Supplementary Fig. 1C) or *SLC25A17* (Supplementary Fig. 2C) in the AD cohort. Next, to predict the contribution of the transcriptome-wide significant genes to the development of hippocampal atrophy accompanying AD, we performed partial least square regression (PLSR) analysis, which is a frequently-used statistical tool in neuroimaging studies of small-scale cohorts (N < 200) to reliably identify relevant variables and estimate their impacts [50, 51]. In addition to the tested genes, aging, sex, and *ApoE4* status, which are strong risk factors associated with brain atrophy [60–62], were also included as critical co-variables in the analysis. By setting hippocampal atrophy rate as the dependent variable, the first three latent factors explained ~80% of variance, and *SLC25A22* expression exhibited top variable importance in the projection (VIP), outweighing *ApoE4* and age (Fig. 3D) and demonstrated, by its weight, a strong negative association with annualized hippocampal loss (Fig. 3E & F) in all three conditions. However, neither *SLC25A10* (Supplementary Fig. 1D–F) nor *SLC25A17* (Supplementary Fig. 2D-F) exhibited strong influence on hippocampal atrophy in AD patients by PLSR analysis. Collectively, these findings indicate the role of hippocampal *SLC25A22* regulation with pathological characteristic of AD and further support an association of *SLC25A22* with the development of AD.

**Table 3:** Demographics of subjects with multiple MRI scans and hippocampal volumetric measurements in the ADNI cohort.

		nonAD (*n* = 164)	AD (*n* = 96)	*p* value
	Age^a^	77.69 ± 0.97	78.58 ± 1.3	0.2761
	Education years^a^	16.55 ± 0.41	15.92 ± 0.56	0.0703
	Sex % female	53.66%	40.63%	0.0425
	*ApoE4*% carrier	22.45%	65.63%	<0.0001
Race	American Indian/Alaska Native	0.61%	0%	0.2142
	Asian	0.61%	2.08%	
	Black or African American	4.88%	0%	
	Other	1.83%	1.05%	
	White	92.07%	96.88%	
AD symptom control medication	None	96.95%	28.13%	<0.0001
	ACHEI	2.44%	7.29%	
	Memantine	0.61%	7.29%	
	ACHEI + memantine	0%	37.5%	
Lifestyle	Alcohol use	3.57%	5.21%	0.7274
	Substance use	1.22%	1.04%	0.8969
	Smoking	42.68%	44.79%	0.7406

^a^Data represented by mean ± 95% CI or percentage.

Two-tailed Student’s t-test were used to compare the difference for quantitative variables, Chi-squared and Fisher’s exact probability tests for qualitative variables.

### Association of susceptibility SLC25 family genes with AD risk

**Fig. 4:**
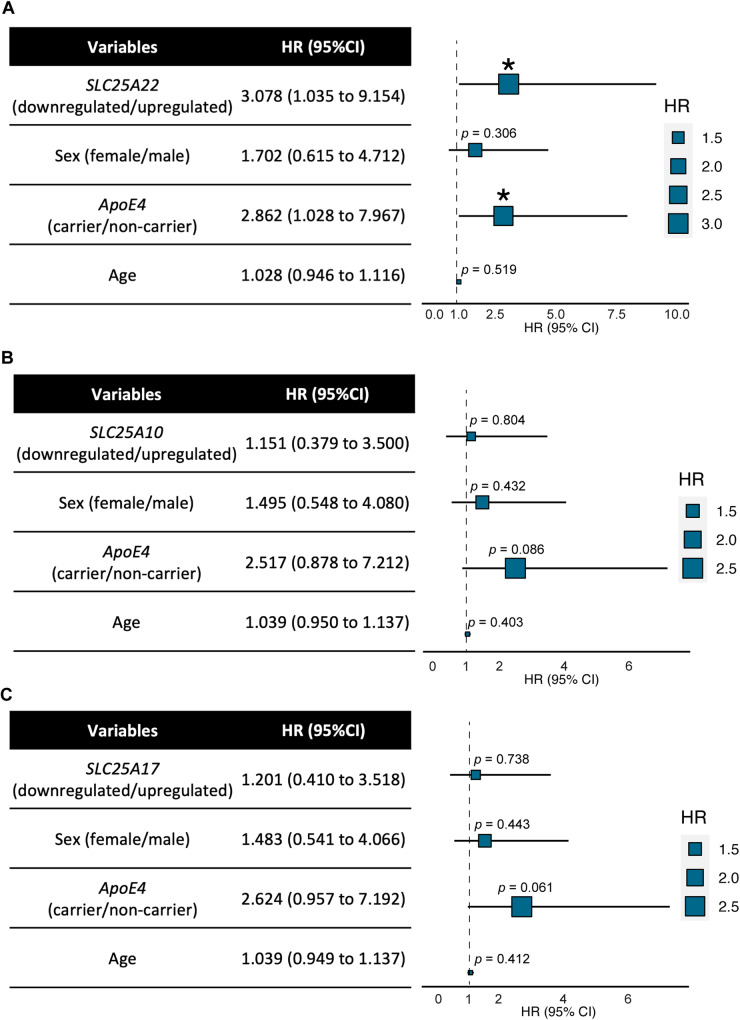
Association of susceptibility *SLC25* genes with the risk of AD. Cox proportional hazards regression models for the association of dementia onset with genetic regulation of hippocampal *SLC25A22* (**A**), *SLC25A10* (**B**), and *SLC25A17* (**C**) within the follow-up period of up to ten years. Sex, *ApoE4* status, and age were included as covariates in multivariable Cox regression. * *p* < 0.05. A total of 124 subjects were included for the analysis. HR: hazard ratio.

To examine whether genetic regulation of hippocampal *SLC25A10*, *SLC25A17*, and *SLC25A22* may promote AD development, a total of 124 subjects in the ADNI cohort with the diagnosis of nonAD and the global clinical dementia rating (CDR) score of 0 at the initial visit as well as yearly CDR evaluations were included in the study. The demographic information of the selected subjects is presented in Table 4. Within the follow-up period of up to ten years, the studied subjects with a change in global CDR score from 0 to 1 or greater were considered “at risk of dementia (event)”. Multivariable Cox proportional hazards regression was performed for the time-event relationship. After adjustment for *ApoE4* status, sex, and age, hippocampal *SLC25A22* downregulation was significantly associated with a faster development of dementia, demonstrated by a hazard ratio (HR) of 3.078 (95% CI:1.035 to 9.154) (Fig. 4A). In contrast, an effect on the speed of dementia onset was not obtained with genetic regulation of *SLC25A10* (Fig. 4B) or *SLC25A17* (Fig. 4C). Therefore, among the three susceptibility genes associated with AD, downregulation of hippocampal *SLC25A22* demonstrates an association with an increased probability of dementia.

**Table 4:** Demographics of subjects with multiple global clinical dementia rating (CDR) evaluation up to ten years in the ADNI cohort.

		CDR < 1 (*n* = 108)^c^	CDR ≥ 1 (*n* = 16)	*p* value
	Age^a,b^	74.15 ± 0.94	75.74 ± 3.13	0.2434
	Education years^b^	16.74 ± 0.53	16.13 ± 1.35	0.4038
	Sex % female	50%	31.25%	0.1611
	*ApoE4*% carrier	26.85%	25%	0.8757
Race	American Indian/Alaska Native	0%	0%	0.2360
	Asian	0.926%	0%	
	Black or African American	7.41%	0%	
	Other	0%	0%	
	White	91.67%	100%	
AD symptom control medication	None	86.11%	87.5%	0.6103
	ACHEI	8.33%	0%	
	Memantine	2.78%	6.25%	
	ACHEI + memantine	2.78%	6.25%	
Lifestyle	Alcohol use	3.77%	0%	0.4339
	Substance use	1.89%	0%	0.5832
	Smoking	41.17%	37.5%	0.7519

^a^Age at initial visit.

^b^Data represented by mean ± 95% CI or percentage.

^c^Throughout the follow-up period of 10 years.

Two-tailed Student’s t-test were used to compare the difference for quantitative variables, Chi-squared, and Fisher’s exact probability tests for qualitative variables.

### Functional annotation of hippocampal gene expression associated with genetic SLC25A22 regulation

**Fig. 5:**
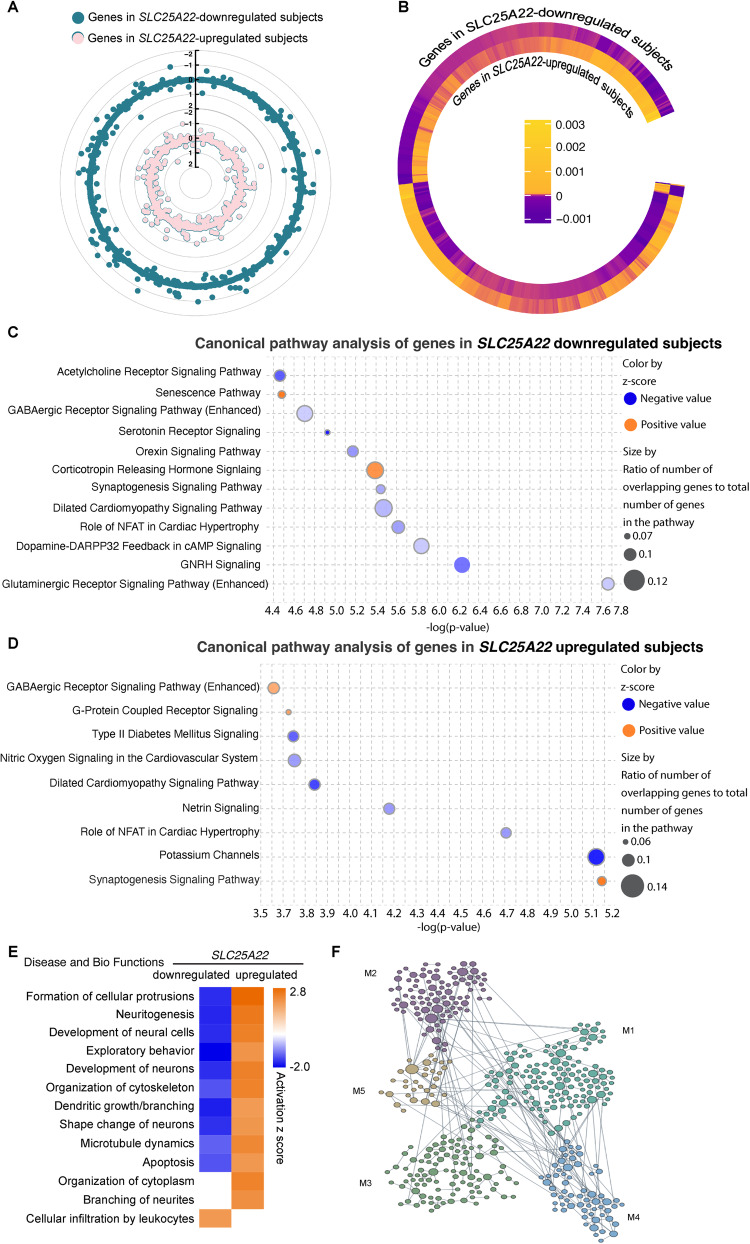
Pathway and network analysis of transcriptomic architecture in *SLC25A22* down- and up-regulated subjects. **A** Regulation of hippocampal genes in *SLC25A22* down- and up-regulated subjects. **B** Genes with reverse z score directions in *SLC25A22* down- and up-regulated subjects. **C**, **D** Canonical pathway analysis of genes in *SLC25A22* downregulated **C** and upregulated **D** subjects. **E** Comparison analysis of genes with opposite z score directions between *SLC25A22* down- and up-regulated subjects. **F** Hippocampal network analysis of genes with opposite z score directions between *SLC25A22* down- and up-regulated subjects in cohesive gene clusters. M1: double-strand DNA stability, M2: developmental growth, M3: lipid metabolism and inflammation, M4: cytosolic transport, M5: regulation of transporter activity.

To interpret the biological significance of the *SLC25A22* gene trait and examine the pattern of transcriptomic regulation in subjects with opposite *SLC25A22* regulation, the tested cohort was clustered into two subsets: *SLC25A22* down- and up-regulated groups, respectively. After comparison of the mean value of all the genes predicted by hippocampal TWAS (Fig. 5A), a total of 622 genes with reversed z score direction between the two groups were identified (Fig. 5B). Genes with predicted values in the *SLC25A22* down- and upregulated groups were subjected to variant loss/gain analysis using Qiagen Ingenuity Pathway Analysis (IPA) software for canonical pathway analysis. Among the downregulated signaling pathways, multiple pathways related to biological processes in the brain, including “glutaminergic receptor signaling pathway”, “dopamine-DARPP32 feedback in cAMP signaling”, “synaptogenesis signaling pathway”, “orexin signaling pathway”, “serotonin receptor signaling”, and “GABAergic receptor signaling pathway” as well as “acetylcholine receptor signaling pathway” were noted. “Senescence pathway” and “corticotropin releasing hormone signaling” topped the upregulated pathways (Fig. 5C). In sharp contrast, “synaptogenesis signaling pathway” and “GABAergic signaling pathway”, which were suppressed in the *SLC25A22* downregulated group, were predicted to be enhanced in subjects with upregulated *SLC25A22* (Fig. 5D). Furthermore, comparison pathway analysis using the identified 622 genes with reversed z score direction between the *SLC25A22* down- and up-regulated groups demonstrated inverted regulation of several pathways related to neuronal function and development, with “formation of cellular protrusions”, “neuritogenesis”, and “development of neural cells” at the top (Fig. 5E). To examine the impact(s) of differentially-regulated genes on biological functions in the context of hippocampal tissues, the 622 genes with reversed z score direction between the *SLC25A22* down- and up-regulated groups were mounted to the hippocampal network in the HumanBase online website (https://hb.flatironinstitute.org) [46] and clustered in multiple modules of biological processes such as “double-strand DNA stability” (M1), “developmental growth” (M2), “lipid metabolism and inflammation” (M3), “cytosolic transport” (M4), and “regulation of transporter activity” (M5) (Fig. 5F and Supplementary Table 2). Those findings support functional and developmental defects of hippocampal neurons in subjects with *SLC25A22* downregulation.

### Selective abundance of SLC25A22 gene expression in human glutamatergic neurons

**Fig. 6:**
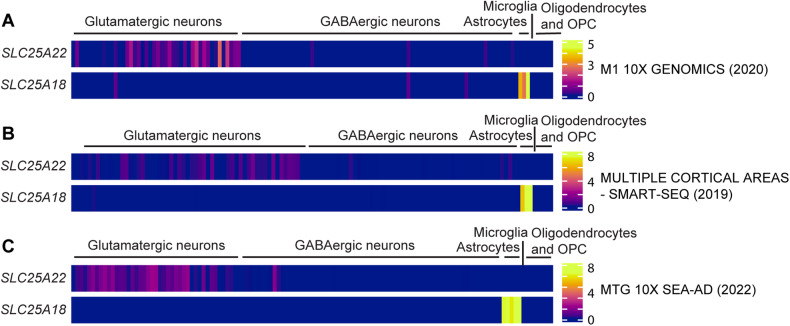
Cell-type enrichment of *SLC25A22* mRNA in human brains. **A**–**C** Heatmaps of *SLC25A22* and *SLC25A18* expression in different brain cell types of single cell sequencing data from **A** M1 10X GENOMICS (2020), **B** MULTIPLE CORTICAL AREAS-SMART-SEQ (2019), **C** MTG 10X Seattle Alzheimer’s Disease Brain Cell Atlas (SEA-AD) (2022). OPC: Oligodendrocyte progenitor cell.

Previous studies have determined two types of mitochondrial glutamate carriers, including SLC25A22 and SLC25A18 located in the inner mitochondrial membrane both designated for glutamate transport [54]. The high degree of biological homology in both their sequences and functions [54] thus raise an interesting question about whether genetic downregulation of *SLC25A22* could be, at least in part, compensated by the unaffected expression of *SLC25A18*. To this end, we sought to examine expression patterns of *SLC25A22* and *SLC25A18* in the human brain at the cell-type resolution using the Allen brain map online tools (https://portal.brain-map.org/atlases-and-data/rnaseq) [47, 48]. In searching for *SLC25A22* and *SLC25A18* expression in the Human M1 10X single-cell sequencing data, we noticed that *SLC25A22* in the brain is predominantly expressed by glutamatergic neurons, which differs from the abundance of *SLC25A18* expression in astrocytes (Fig. 6A). Similar patterns of *SLC25A22* and *SLC25A18* expression in the human brain were determined in the SMART-SEQ (Fig. 6B) and Seattle Alzheimer’s Disease Brain Cell Atlas (SEA-AD) - Spatial transcriptomics - MERFISH studies (Fig. 6C). The distinct expression patterns of *SLC25A22* and *SLC25A18* indicate glutamatergic neuronal vulnerability to genetic downregulation of *SLC25A22* and further correlate with our functional annotation of hippocampal genes showing deficits in neuronal function and development in subjects with genetic downregulation of *SLC25A22*. Of note, a study done in rats has determined higher expression of SLC25A22 in astrocytes than in neurons [63]. Furthermore, although mRNA levels do not always correlate with protein expression, the results of selective abundance of *SLC25A22* mRNA in human neurons together with reported enrichment of SLC25A22 in mouse neurons [64, 65] seem to implicate a species-related difference in SLC25A22 expression.

## Discussion

In contrast to the Mendelian inheritance pattern of familial Alzheimer’s disease (AD), the contribution of genetic components to the etiopathogenesis of late-onset Alzheimer’s disease (AD), especially in patients without a clear history of transmission, remains unclear [66]. In recent years, several powerful tools including tissue-specific transcriptome-wide association studies (TWAS) and Mendelian randomization (MR) have been developed to assist in the functional interpretation of genome-wide association studies (GWAS), which disentangle the association of genetic variants within genetically complex diseases such as late-onset AD [32, 67, 68]. The mitochondrial solute carrier family (SLC25), consisting of 53 members, constitutes crucial transporters for the exchange of a plethora of small molecules between mitochondria and the cytosol and/or other cytosolic organelles [19, 22]. Consistent with their importance to mitochondrial function, defects within the SLC25 members have been linked to mitochondrial dysfunction in cancers as well as in a diverse spread of neuro-developmental and degenerative disorders [22, 69–71]; however, the genetic variability of *SLC25*s and their phenotypic consequences in AD have never been comprehensively investigated. In this study, we conducted hippocampal TWAS analysis to predict gene expression of the *SLC25* family and their association(s) with AD using GWAS summary data from two extensive cohorts of AD patients and individual WGS data from the ADNI cohort. The findings revealed a significant association between the genetic regulation of *SLC25A10, SLC25A17*, and *SLC25A22*, and the risk of developing AD. Further neuroimaging studies indicated a strong reverse relationship between *cis*-regulation of *SLC25A22* and hippocampal atrophy rate. Notably, our partial least square regression (PLSR) analysis using the ADNI cohort weighed *SLC25A22* over well-characterized risk factors for AD and AD-associated hippocampal atrophy such as sex (female), aging, and *ApoE4* status [60–62, 72, 73] in disrupting hippocampal volumetric integrity. Therefore, these results support *SLC25A22* as a susceptibility gene for AD and further connect *SLC25A22* downregulation to hippocampal vulnerability in a subset of AD patients. It should be noted that in addition to the hippocampus, other brain regions such as the amygdala, cingulate gyrus, temporal and frontal cortexes, and others are also affected in AD [74]. Taking into consideration the abundance of *SLC25A22* in glutamatergic neurons, *SLC25A22* downregulation may also be associated with accelerated decay with disease progression in these brain regions, which warrants further investigation.

In view of our results, it would be of paramount interest to explore the potential mechanisms underlying the association of *SLC25A22* with AD. *SLC25A22*-encoded protein [SLC25A22, also known as glutamate carrier 1 (GC1)] together with SLC25A18 [also known as mitochondrial glutamate carrier 2 (GC2)] are the two carriers for the mitochondrial transport of glutamate. Our analysis of the single-cell sequencing data from the Human M1 10X single-cell sequencing, SMART-SQ and SEA-AD data indicates an abundance of *SLC25A22* expression in glutamatergic neurons and selective *SLC25A18* expression in astrocytes. These findings not only suggest the limited capacity of SLC25A18 to compensate for neuronal loss of SLC25A22, but also raise a critical scientific question regarding the yet-underappreciated role of SLC25A22 in neuronal glutamate metabolism. It is well-established that glutamate, the primary excitatory neurotransmitter, is predominantly cleared by astrocytes after its release into the synaptic cleft [75]. In recent years, the role of neurons in glutamate metabolism has gained increasing attention, and experimental evidence suggests that part of the glutamate released from synapses can be sent back to neurons, probably via glutamate transporter 1 encoded by *SLC1A2* [76]. In addition to its return into synaptic vesicles for reuse, the reabsorbed glutamate also can be used by mitochondria to fuel oxidative phosphorylation (OXPHOS) for ATP generation [77]. Previous studies have highlighted a key role of mitochondrial glutamate/aspartate antiporter, SLC25A12, in importing glutamate into mitochondria to complete the malate-aspartate shuttle (MAS) and energize mitochondria in neurons as well as other types of cells in the brain [78–80]. Additionally, in light of its function as a mitochondrial glutamate/H^+^ symporter, a significant contribution of SLC25A22 to mitochondrial glutamate transport and subsequent bioenergetic regulation of neurons is emerging [65, 81], which supports our hypothesis that lowered expression of *SLC25A22*, even to a small degree, compromises neuronal and mitochondrial glutamate metabolism, which causes energy deficiency and cytosolic glutamate overburden and predisposition to AD. Intriguingly, a recent study reported that activated expression of neuron-enriched mitochondrial proteins, including SLC25A22 in astrocytes promotes the conversion of astrocytes to neurons in vitro [64]. These interesting findings not only underscore the significance of SLC25A22 in the homeostatic regulation of neuronal function but also implicate the potentially deleterious impact of *SLC25A22* downregulation on neuronal regeneration in neurodegenerative disorders, including AD. It should be noted that consistent with the importance of SLC25A22 to mitochondrial and neuronal fitness, exonic mutations in *SLC25A22* have been linked to multiple developmental disorders such as thiamine metabolism dysfunction syndrome 4, combined D-2- and L-2-hydroxyglutaric aciduria, mitochondrial phosphate carrier deficiency, and early infantile epileptic encephalopathy [26–31]. In our current study, in contrast to exonic mutations in *SLC25A22* that are associated with lethal developmental disorders, the identified AD-associated mutations are located in the non-coding regions of *SLC25A22*. Although the current study on intronic mutations is still in an early stage, emerging evidence highlights the potential impacts of mutations in the noncoding regions on gene expression or splicing through various pathways, such as the short-distance regulation of the efficacy of promoters and/or enhancers, and long-distance modulation of intron-promoter 3D interactions [82, 83]. Therefore, it is possible that the AD-associated *SLC25A22* polymorphism has a relatively weak but persistent deleterious impact on gene expression, culminating in severe pathological consequences with age.

Subjects with *SLC25A22* downregulation also demonstrated altered transcriptomic architecture with changes in the genetic regulation of a collection of genes including *ABLIM1*, which encodes actin-binding LIM protein 1, *ABRA*, which encodes actin-binding Rho-activating protein, *VAMP5*, which encodes vesicle-associated membrane protein 5, *STX3*, which encodes syntaxin 3, *NLGN1*, which encodes neuroligin 1, and many others. These genes were mounted to pathways related to neuronal function and development such as “formation of cellular protrusions”, “neuritogenesis”, and “development of neural cells”, as well as “dendritic growth/branching” and “shape change of neurons”. Although data from a larger population is needed to confidently establish the link between *SLC25A22* and all or some of these genes, current results seem to add further supportive evidence to the deleterious influence of *SLC22A22* downregulation on neuronal development and function. Indeed, it should be remembered that the TWAS-predicted effects of regulatory variants indicate the genetic regulation of selected gene(s) but may not fully represent actual gene expression and, to a further extent, actual protein expression in each individual [34]. To this end, the impact of genetic *SLC25A22* due to regulatory variants on mitochondrial function and neuronal glutamate metabolism requires further in-depth investigation and validation.

Lastly, this study indicates that mitochondrial factors may independently contribute to AD. Previous studies on mitochondrial dysfunction in brain aging and AD have overwhelmingly focused on mitochondrial dysfunction in response to the aging process, AD-associated pathological molecules, and systemic factors with disease progression [7, 9–13, 84], thus underscoring the contributing role of mitochondrial dysfunction to AD development. However, a key scientific question about whether mitochondria play a proactive role in the etiopathogenesis of AD or act as passive responders to AD-associated toxic proteins remains unaddressed. To date, evidence of the mitochondrial role as an initiator of AD is emerging. Multiple heteroplasmic mitochondrial DNA (mtDNA) mutations have been reported in AD patients [85–89]. Previous studies have shown that transferring platelet mtDNA from AD patients into cells with depletion of endogenous mtDNA promotes AD-like changes [90, 91], these findings as well as the association of mtDNA haplogroups with AD risk [92] add credit to the hypothesis of mitochondria-related mode of transmission in the development of this neurodegenerative disorder. In addition to mtDNA mutations, our findings of nuclear DNA (nDNA)-encoded *SLC25*-related susceptibility genes of AD yield new insight into the interaction between genetic variability and mitochondria in the development of dementia. Because the vast majority of mitochondria-associated proteins are encoded by nuclear genes [93], increased attention to the connection between mutations in mitochondrial protein-encoding nuclear genes and mitochondrial deficits will broaden our view of inherited mitochondrial dysfunction in the development of AD and further strengthen the mitochondrial cascade hypothesis of this neurodegenerative disorder [10, 94, 95]. Furthermore, increasing evidence implicates somatic genetic [96, 97] and epigenetic [98, 99] factors in dementia. To this end, in addition to hereditary mutations, the contribution of somatic mutations and epigenetic regulation of mitochondrial genes to AD risk also merits further investigation.

Several limitations of the current study should also be noted. Although we established the association of transcriptome-wide significant *SLC25* family members, including *SLC25A10*, *SLC25A17*, and *SLC25A22* with AD, further phenotypic association of *SLC25* family genes with hippocampal volumetric loss was conducted using a cohort of a relatively smaller size. Moreover, due to the limitations of the reference transcriptomic data, the hippocampal expression of only forty-one out of the total of fifty-three *SLC25* family genes was predicted and used in this study. Furthermore, genetic data was collected primarily from subjects with European ancestry. These caveats warrant the need for future validation of our findings through the analysis of neuroimaging and genetic datasets with larger sample size and broader diversity. Of note, in the current study, we primarily focused on the association of *SLC25A22* regulation with the risk of late onset AD. A related question is whether *SLC25A22* polymorphism may also play a role in the development of early-onset AD. It is well-documented that the etiopathogenesis of early-onset AD is closely related to autosomal dominant mutations in *PSEN1*, *PSEN2*, and *APP* [100]. These genetic alterations lead to an earlier onset and accelerated progression of the disease. We cannot fully exclude the contribution of *SLC25A22* polymorphism to early onset AD, at least, in a subset of patients although its contribution, if exists, might be relatively weak given the genetic penetrance of the aforementioned early onset-AD-related genetic risks. This outstanding question warrants further investigation of the association of hippocampal *SLC25A22* regulation with phenotype development in patients with early-onset AD and, to a further extent, in patients with other types of AD-related dementia (ADRD) such as Lewy body dementia and Down syndrome, which also demonstrate hippocampal lesions and AD-like cognitive deficits [101, 102].

In summary, our findings identified the association of hippocampal *SLC25* genes, including *SLC25A10*, *SLC25A17*, and *SLC25A22* with AD risk through TWAS-predicted hippocampal gene expression. Further gene-to-disease trait assessment supports the influence of hippocampal *SLC25A22* downregulation on hippocampal lesions in AD and the transition from non-demented to AD. In contrast, hippocampal *SLC25A22* upregulation is protective against AD. Therefore, we conclude that genetic variability of the identified *SLC25* family genes, especially *SLC25A22* contribute to AD. Further investigation on *SLC25A22* in experimental and clinical settings holds promise to deepen our understanding of the mitochondrial pathway of AD and advance the development of strategies targeting this AD-susceptibility gene for the diagnosis and early prevention of this neurodegenerative disorder.

### Supplementary information

Supplemental figures and tables

## Data Availability

The data of this study is available from the corresponding authors upon request with additional approval.

## Code availability

The analyses were conducted using standard code that are available in the software instruction page and can be provided by the corresponding author upon request. All software used in this study is freely available online.
