# Supplementary material for: Hippocampal transcriptome-wide association study and pathway analysis of mitochondrial solute carriers in Alzheimer’s disease
Source: Transl Psychiatry. 2024 Jun 10;14:250. doi: 10.1038/s41398-024-02958-0 (PMC11164935; doi:10.1038/s41398-024-02958-0)
Supplement: Supplementary file 1 — Supplemental figures and tables [file 41398_2024_2958_MOESM1_ESM.docx]

**Supplementary figures and tables:**

**
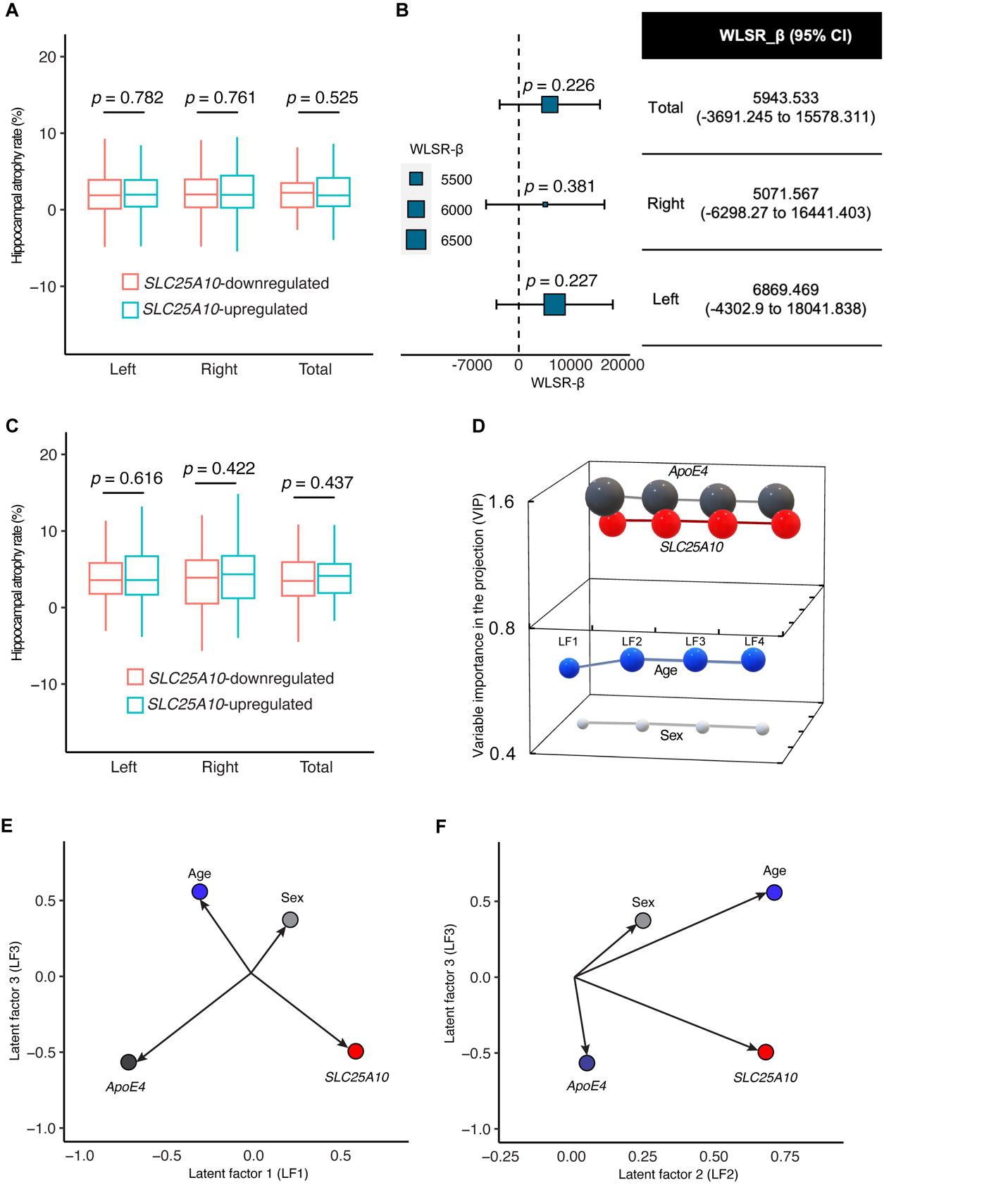
**

**sFig. 1. Analysis of the relationship between hippocampal *SLC25A10* genetic regulation and hippocampal atrophy rate. (A)** Comparison of hippocampal atrophy rate between *SLC25A10* down- and up-regulated subjects in the AD and nonAD combined cohort. Two-tail student t-test. *SLC25A10* downregulated *n* = 150, *SLC25A10* upregulated *n* = 110. Data is represented as mean ± 95% CI. **(B)** Weighted least square regression (WLSR) analysis for the correlation between hippocampal atrophy rate and the effect size of *SLC25A10* genetic regulation. *SLC25A10* downregulated *n* = 150, *SLC25A10* upregulated *n* = 110. **(C)** Comparison of hippocampal atrophy rate between *SLC25A10* down-and up-regulated subjects in AD patients. Two-tail student t-test. *SLC25A10* downregulated *n* = 54, *SLC25A10* upregulated *n* = 42. Data is represented as mean ± 95% CI. **(D-F)** Partial least square regression (PLSR) analysis in the AD cohort. The annualized hippocampal atrophy rate was set as the dependent variable. *SLC25A10*, age, sex, and *ApoE4* status were input as covariables in the analysis. **(D)** Variance importance in the projection (VIP) of *SLC25A10,* age, sex, and *ApoE4*. **(E&F)** GGraphics of latent factors 1 and 3 **(E)** as well as 2 and 3 **(F)**.

**
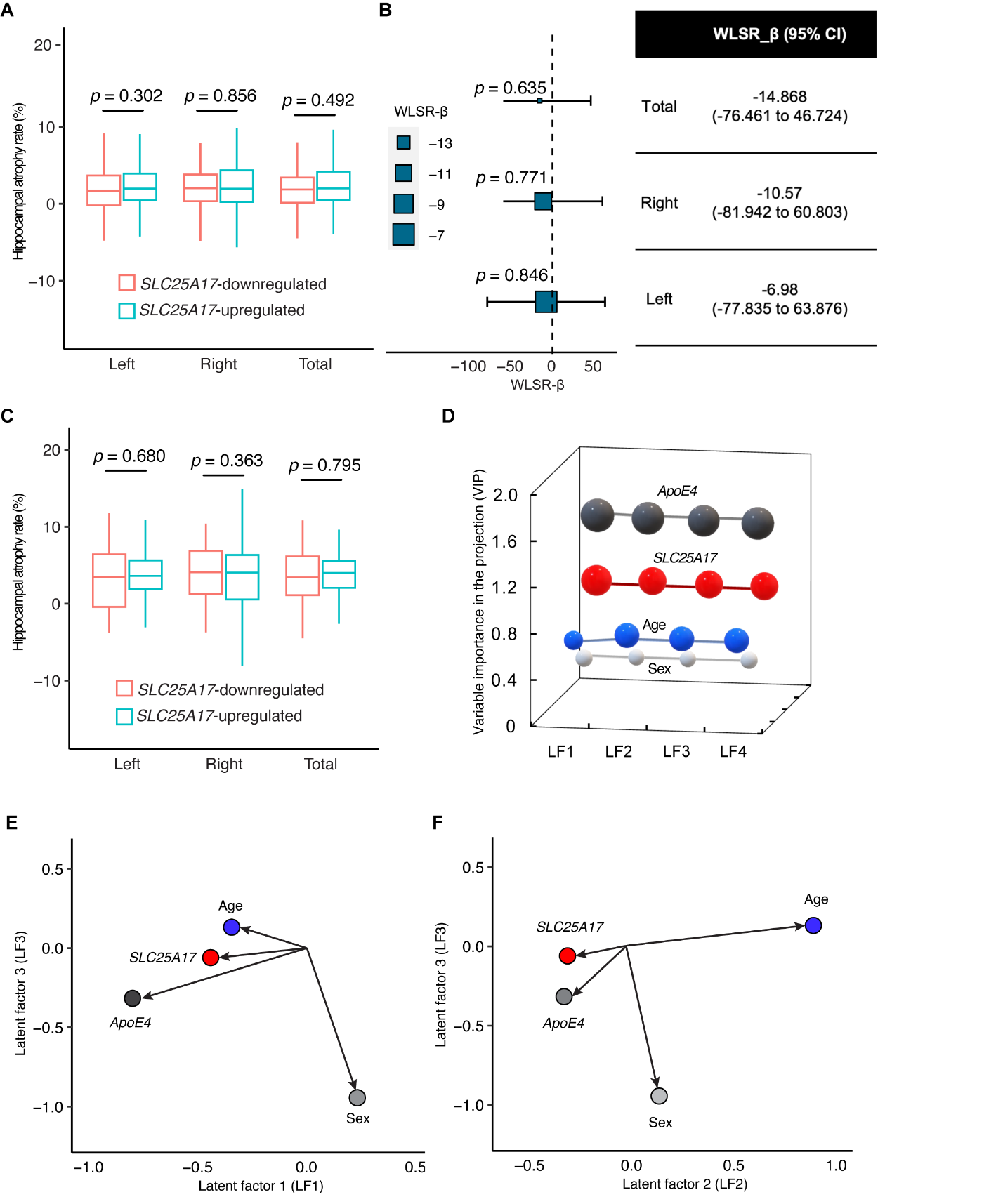
**

**sFig. 2. Analysis of the relationship between hippocampal *SLC25A17* genetic regulation and hippocampal atrophy rate. (A)** Comparison of hippocampal atrophy rate between *SLC25A17* down- and up-regulated subjects in the AD and nonAD combined cohort. Two-tail student t-test. *SLC25A17* downregulated *n* = 86, *SLC25A17* upregulated *n* = 174. Data is represented as mean ± 95% CI. **(B)** Weighted least square regression (WLSR) analysis for the correlation between hippocampal atrophy rate and the effect size of *SLC25A10* genetic regulation. *SLC25A22* downregulated *n* = 86, *SLC25A10* upregulated *n* = 174. **(C)** Comparison of hippocampal atrophy rate between *SLC25A17* down- and up-regulated subjects in AD patients. Two-tail student t-test. *SLC25A17* downregulated *n* = 29, *SLC25A17* upregulated *n* = 67. Data is represented as mean ± 95% CI. **(D-F)** Partial least square regression (PLSR) analysis in the AD cohort. The annualized hippocampal atrophy rate was set as the dependent variable. *SLC25A17*, age, sex, and *ApoE4* status were input as covariables in the analysis. **(D)** Variance importance in the projection (VIP) of *SLC25A17,* age, sex, and *ApoE4*. **(E-F)** GGraphics of latent factors 1 and 3 **(E)** as well as 2 and 3 **(F)**.

**sTable 1. Cerebellar *SLC25A10*, *SLC25A17* and *SLC25A22* association with AD**

| Gene symbol | Discovery cohort 1 | | Discovery cohort 2 | | | ADNI cohort | | | Meta analysis | |
| --- | --- | --- | --- | --- | --- | --- | --- | --- | --- | --- |
|  | z score | *p* value | z score | *p* value | z score | | *p* value | Combined effect direction | | Combined *p* value |
| *SLC25A10* | -1.1591854 | 2.46E-01 | -0.8571851 | 0.3917767 | -0.572931 | | 5.67E-01 | - | | 0.27171478 |
| *SLC25A17* | 1.61838752 | 1.06E-01 | 0.75349455 | 0.45152768 | 1.79477209 | | 7.27E-02 | + | | 0.02700505 |
| *SLC25A22* | -1.1090724 | 2.67E-01 | 0.46454691 | 0.64246971 | -0.8791767 | | 3.79E-01 | - | | 0.21901101 |

| Node | Top terms (Max 10) | Q val | Genes | Terms |
| --- | --- | --- | --- | --- |
| **M1** | double-strand break repair | 0.0167 | 141 | 60 |
|  | DNA double-strand break processing | 0.0167 |  |  |
|  | DNA repair | 0.0286 |  |  |
|  | regulation of DNA repair | 0.0286 |  |  |
|  | double-strand break repair via single-strand annealing | 0.0286 |  |  |
|  | DNA double-strand break processing involved in repair via single-strand annealing | 0.0286 |  |  |
|  | DNA recombination | 0.0330 |  |  |
|  | lamellipodium assembly | 0.0346 |  |  |
|  | pyrimidine nucleotide metabolic process | 0.0346 |  |  |
| **M2** | protein localization to chromosome | 0.0393 | 88 | 45 |
|  | G-protein coupled receptor signaling pathway | 0.0248 |  |  |
|  | response to hormone | 0.0248 |  |  |
|  | positive regulation of multicellular organism growth | 0.0284 |  |  |
|  | multicellular organism growth | 0.0286 |  |  |
|  | regulation of multicellular organism growth | 0.0286 |  |  |
|  | regulation of developmental growth | 0.0382 |  |  |
|  | response to steroid hormone | 0.0408 |  |  |
|  | organic cation transport | 0.0408 |  |  |
|  | oligosaccharide metabolic process | 0.0447 |  |  |
|  | response to glucocorticoid | 0.0472 |  |  |
| **M3** | chemokine production | 0.0286 | 98 | 50 |
|  | regulation of chemokine production | 0.0286 |  |  |
|  | regulation of interleukin-1 production | 0.0286 |  |  |
|  | regulation of interleukin-1 beta production | 0.0286 |  |  |
|  | interleukin-1 beta production | 0.0296 |  |  |
|  | low-density lipoprotein particle clearance | 0.0330 |  |  |
|  | interleukin-1 production | 0.0330 |  |  |
|  | triglyceride homeostasis | 0.0408 |  |  |
|  | acylglycerol homeostasis | 0.0408 |  |  |
|  | plasma lipoprotein particle clearance | 0.0494 |  |  |
| **M4** | retrograde transport, endosome to Golgi  cytosolic transport  endosomal transport  glycosaminoglycan metabolic process  drug transmembrane transport  aminoglycan metabolic process  organic anion transport  regulation of epithelial to mesenchymal transition  drug transport  organelle localization | 0.0330  0.0517  0.0561  0.0564  0.0597  0.0644  0.0704  0.0799  0.0850  0.0904 | 62 | 15 |
| **M5** | regulation of ion transmembrane transporter activity  regulation of transmembrane transporter activity  regulation of transporter activity  negative regulation of cell migration  regulation of epithelial cell proliferation  regulation of ion transmembrane transport  regulation of epithelial cell migration  negative regulation of cell motility  negative regulation of cellular component movement | 0.0630  0.0632  0.0644  0.0817  0.0817  0.0817  0.0850  0.0850  0.0862 | 32 | 19 |

**sTable 2. Clusters of biological functions identified by hippocampal network analysis**
